# Supplementary material for: Oxygen-Sensitive Metalloprotein Structure Determination by Cryo-Electron Microscopy
Source: Biomolecules. 2022 Mar 12;12(3):441. doi: 10.3390/biom12030441 (PMC8945911; doi:10.3390/biom12030441)
Supplement: Supplementary file 1 [file biomolecules-12-00441-s001.zip › biomolecules-1592467-supplementary.pdf]

biomolecules

MDPI

Supplementary Materials

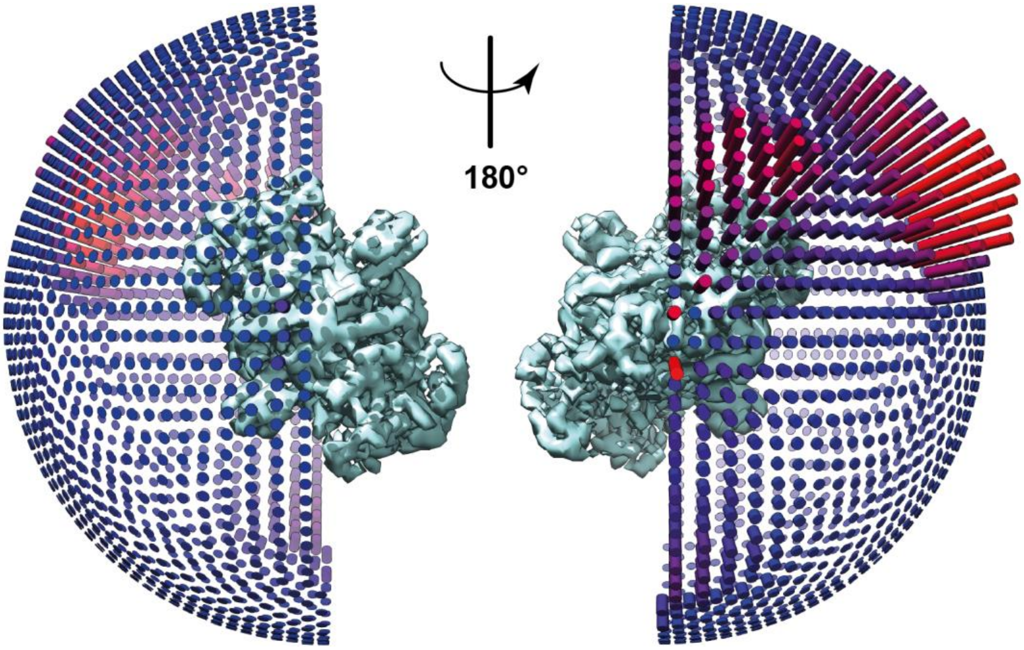

Figure S1. Angular distribution of the particles used for the final PFOR reconstruction. The final round of refinement (cycle 23) used an angular step of 0.4687°. Yet, data from cycle 19 (angular step of 0.9375°) are displayed for better clarity. PFOR reconstruction is displayed in the middle of each half sphere (light blue).

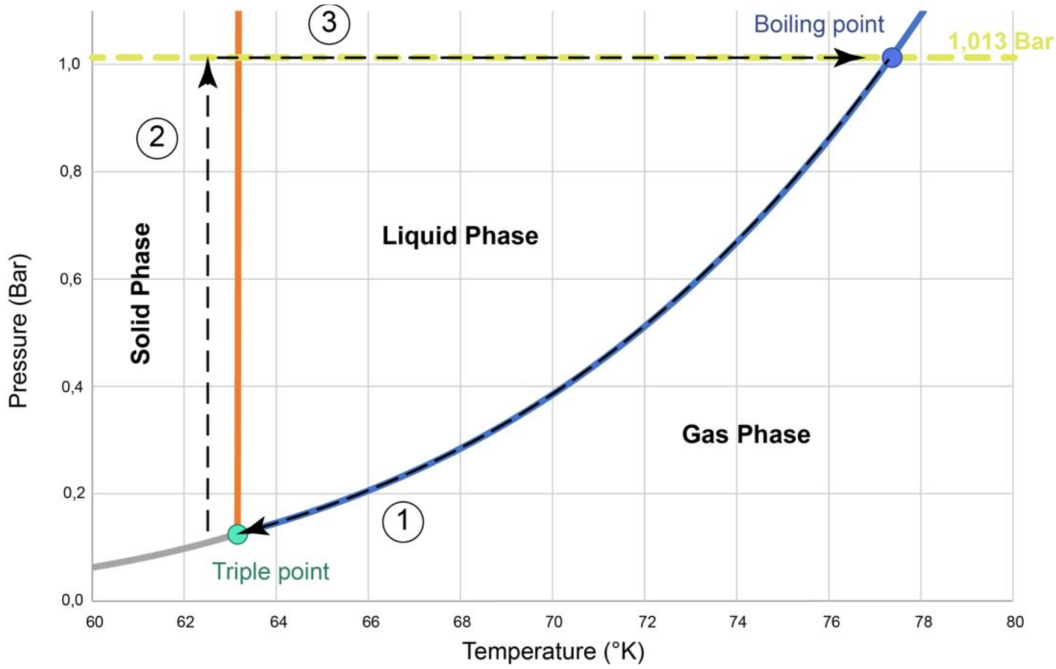

Figure S2. Nitrogen phase diagram. Blue solid line: phase boundaries between gas and liquid nitrogen [1]. Gray solid line: phase boundaries between gas and solid nitrogen. Orange solid line: phase boundaries between solid and liquid nitrogen. Yellow dashed line: atmospheric pressure at 1,013 Bar. The blue and green spheres indicate the Boiling point and the Triple point respectively. The three black dashed lines indicate the approximate path that the nitrogen will follow through the phase diagram: (1) inside the glovebox airlock; (2) at the end of the vacuum cycle when the nitrogen is entered in the glovebox; (3) during the system heating up upon cryo-EM grids flash-cooling.

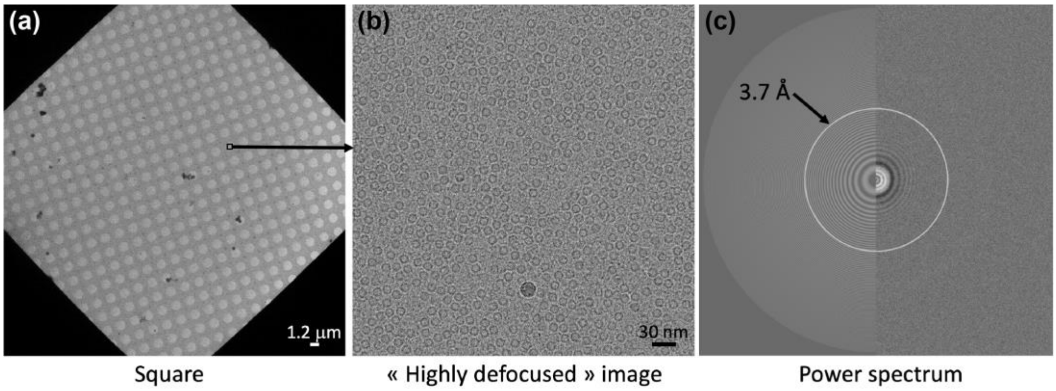

Figure S3. Apoferritin data collection on the Glacios. (**a**) View of a square of the anaerobic cryo-EM grid of apoferritin. (**b**) Example of an image acquired using the K2 (the 60 frames have been re-aligned and averaged as described in the Material and Methods part). Highly defocused image to facilitate visualization of the particles. (**c**) Theoretical (left) and calculated (right) power spectrum of the "highly defocused" image.

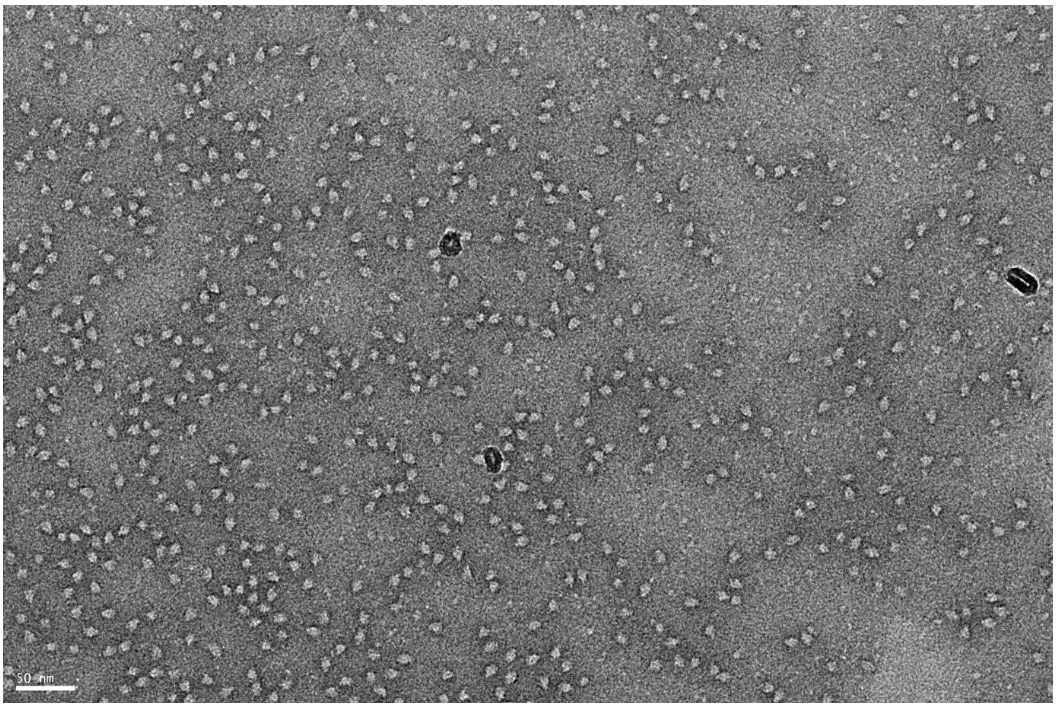

Figure S4. Example of negative stain images of PFOR (magnification x 30000).

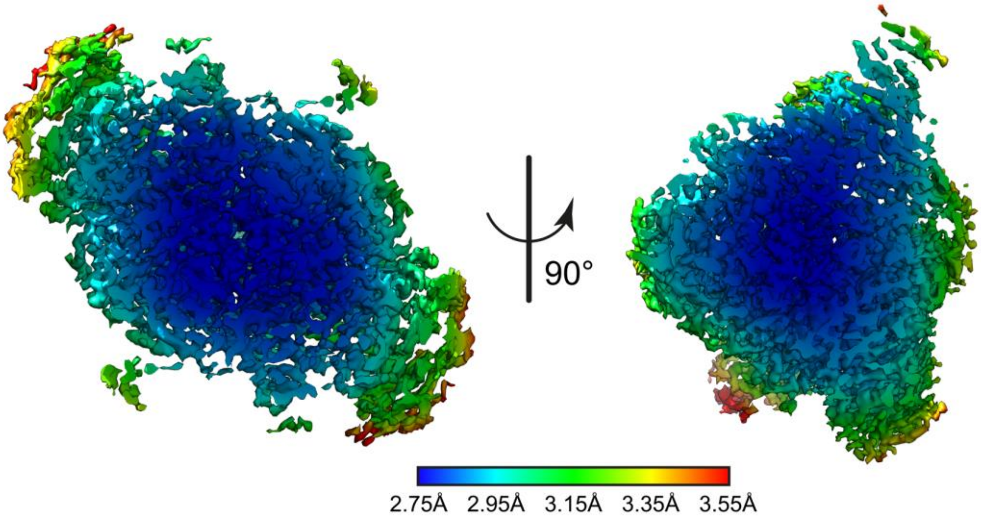

Figure S5. Cross section of the final PFOR reconstruction colored according to the local resolution: from dark blue (2.75 Å) to red (3.55 Å), contoured at 2.7 σ. The same orientation as in Figure 4d has been used.

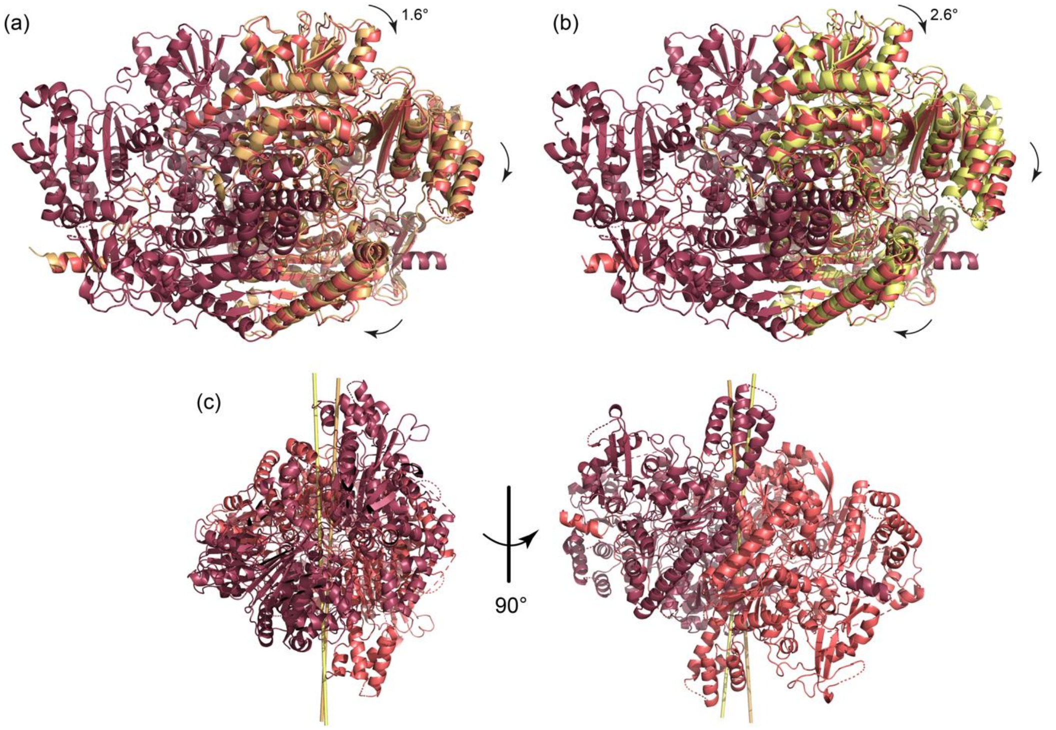

Figure S6. Comparison between the *D. africanus* PFOR cryo-EM model (chain A and chain B colored in dark-red and salmon, respectively) and the X-ray crystallography structure. (**a**) Comparison with *D. africanus* X-ray structure (PDB code: 1B0P; only chain B is displayed and colored in light orange). The two structures are superposed on chain A and the view is oriented along the rotation axis. The curved arrows indicate the direction of the rotation. (**b**) Comparison with *M. thermoacetica* PFOR X-ray structure (PDB code: 6CIN; only chain B is displayed and colored in yellow). The two structures are superposed on chain A and the same view as (a) is used. (**c**) Two orientations of the PFOR cryo-EM model with the rotation axis between this structure and *D. africanus* or *M. thermoacetica* X-ray structure displayed as light orange and yellow cylinders, respectively.

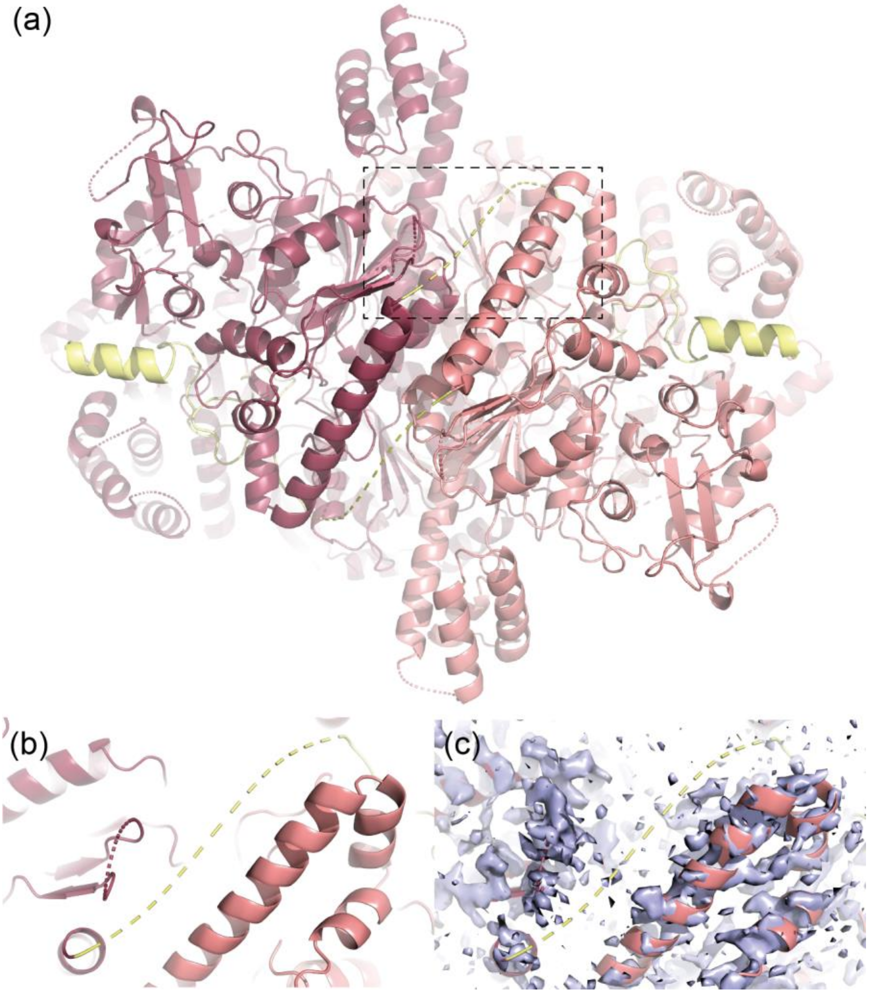

Figure S7. Dimer of the PFOR cryo-EM model: chains A and B are colored in dark-red and salmon, respectively, and domains VII are colored in yellow. Dashed lines indicate missing residues, without any matching density. Close view of the missing residues corresponding to the beginning of domain VII located by the dashed black rectangle in (**a**) and showed (**b**) without and (**c**) with cryo-EM density display at 4 σ.

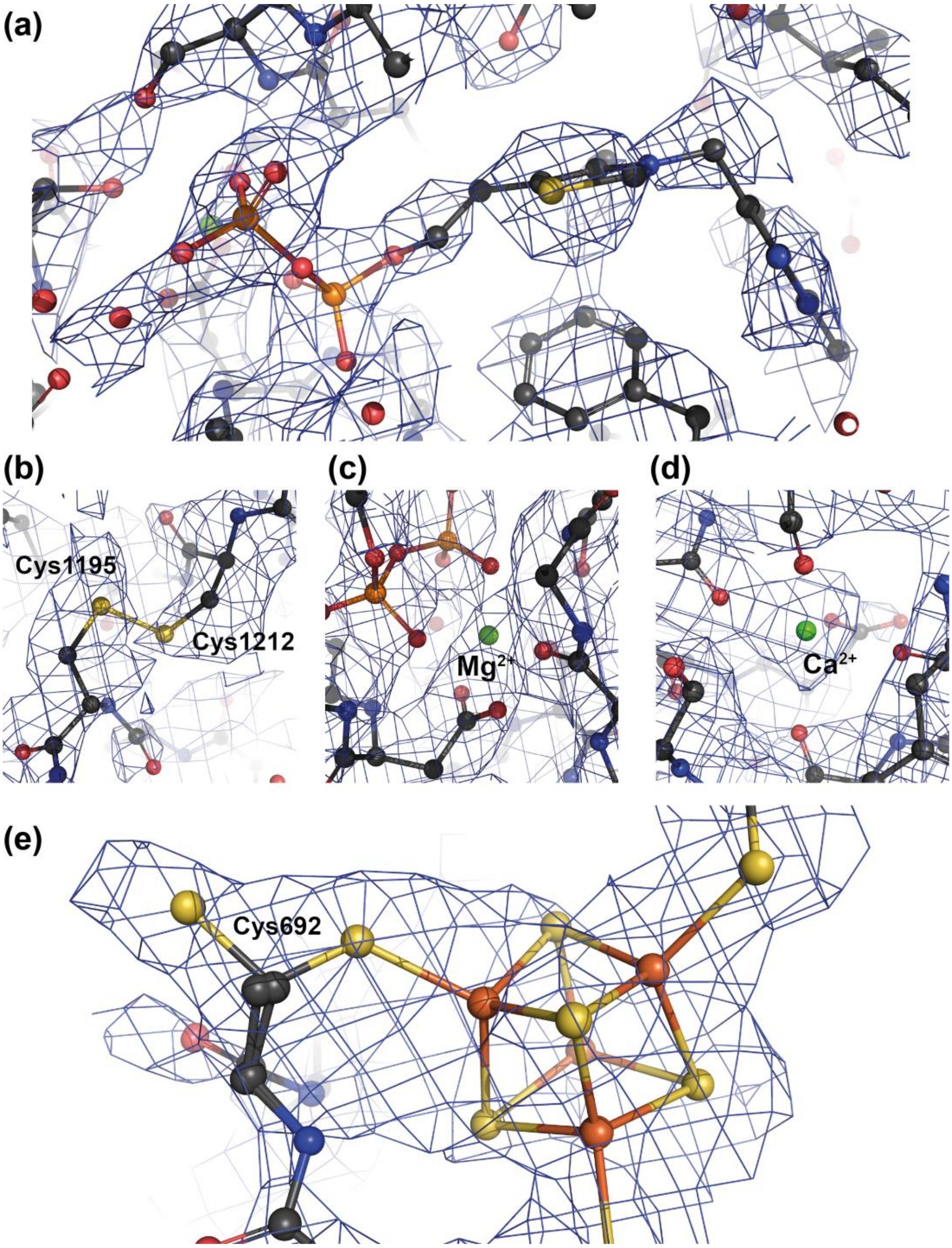

Figure S8. Cryo-EM PFOR density shown at 4 σ of (**a**) the TPP molecule in its binding pocket; (**b**) disulfide bridge between Cys1195 and Cys1212; (**c**) Mg^{2+} ion colored in dark green; (**d**) Ca^{2+} ion colored in green; (**e**) the distal [Fe_4S_4] cluster with an alternative conformation visible for Cys692 side chain.

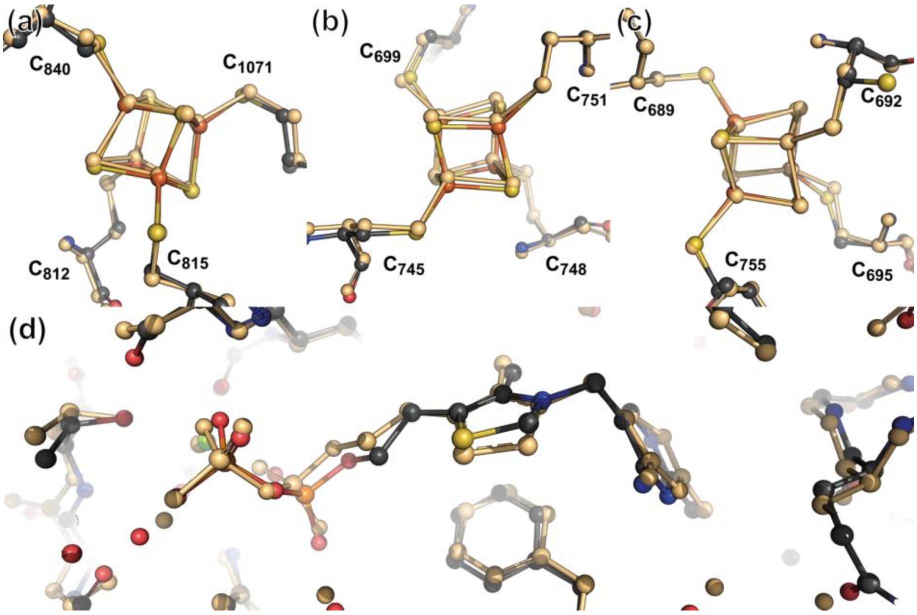

Figure S9. Comparison of the cryo-EM and crystal PFOR structures. Close view of the (**a**), proximal (**b**), median and (**c**) distal [Fe_4S_4] clusters and of the (**d**) TPP molecule. Atoms of the cryo-EM PFOR structure are colored as follow: carbon, black; nitrogen, blue; oxygen, red; sulfur, yellow; iron, brown; phosphorus, orange. Atoms of the crystal PFOR structure are all colored in light orange (PDB code: 1B0P).

1. Engineering ToolBox Nitrogen-Thermophysical Properties. Available online: https://www.engineeringtoolbox.com/nitrogen-d_1421.html (accessed on: 12/01/2021).
